# Water and sanitation in urban India

**DOI:** 10.2471/BLT.24.020824

**Published:** 2024-08-01

## Abstract

Two of the world’s biggest sanitation initiatives are approaching their 10-year anniversaries, offering insights into challenges faced worldwide. Gary Humphreys reports.

For Mahreen Matto, an urban water and sanitation management expert who works at the National Institute of Urban Affairs in Delhi, India’s urban sanitation problem, for all its complexity, can be simply stated. “We generate more wastewater than we can treat,” she says.

Wastewater, as Matto points out, can include domestic, commercial and industrial wastewater that ranges from ‘black’ water containing faecal matter and urine to ‘grey’ water that comes from sources other than toilets, such as sinks, showers, bathtubs and laundry machines.

“According to the 2021 report published by the CPCB (Central Pollution Control Board – India’s principal regulatory body for environmental protection and pollution control activities) – only 20.2 million of the 72.3 million litres of wastewater produced by the roughly 500 million people living in urban India every day, around 28%, was actually treated,” Matto says, adding that the rest finds its way into the environment, including rivers and other water bodies.

Though disquieting, such low levels of wastewater treatment are not unusual. “In the absence of robust, disaggregated data, the overall picture is by no means clear,” says Kate Medlicott, a sanitation and waste expert at the World Health Organization (WHO), “but it is estimated that only around 58% of domestic wastewater (urban and rural) is safely treated, and this average conceals significant differences between high-income and low-income countries where the treatment levels can be much lower,” 

And the problem is only getting worse. “As urban centres grow, sewerage systems are increasingly unable to cope, exposing populations to frequent outbreaks of diseases including typhoid, viral hepatitis and cholera,” Medlicott explains, adding that sewerage systems also provide ideal environments for the emergence and spread of antimicrobial-resistant bacteria. 

According to the WHO/United Nations Children’s Fund (UNICEF) Joint Monitoring Programme for Water Supply, Sanitation and Hygiene (JMP), which was established in 1990 to monitor and report on the progress of water supply and sanitation services worldwide, the lack of access to safely managed sanitation causes 1.4 million preventable deaths annually.

India’s response to the problem has been two-fold and nationwide: the Swachh Bharat Mission (SBM), launched in October 2014 with the aim of revolutionizing sanitation and waste management across the nation; and the Atal Mission for Rejuvenation and Urban Transformation (AMRUT), launched in June 2015 with the aim of improving urban infrastructure in 500 cities, including sewerage and water supply systems.

The SBM is divided into two parts, one targeting rural communities (SBM Gramin), and the other, cities (SBM Urban (SBM-U). Both have focused on eradicating open defecation while ensuring ‘scientific’ disposal of waste.

“[Worldwide] around 58% of domestic wastewater is safely treated.”

Kate Medlicott

The SBM is credited with significantly improving India's sanitation landscape, notable achievements including the construction of over 100 million toilets and a dramatic reduction in open defecation (OD – defecating away from sanitation facilities) driven by nationwide educational campaigns, and the introduction of open defecation free (ODF) certification for cities based on annual third-party assessment.

The 2023 JMP reports that the number of people practicing open defecation in India had decreased to around 75 million that year, down from around 500 million in 2014.

The SBM’s impact on health has yet to be documented, but a recent WHO estimation study indicated that more than 300 000 deaths (from diarrhoea and protein-energy malnutrition) had been averted between 2014 and October 2019 as a result of the SBM Gramin initiative.

The health impact of the SBM-U initiative also awaits elucidation, but there has been significant progress in the distribution of household latrines, 6.6 million of which had been constructed by 2020, meeting a key SBM-U target. 

The target for 100% scientific disposal of waste has proved harder to meet, partly because of the lack of sewerage and faecal sludge infrastructure and management services.

“We have around 4700 cities, of which only about 400 have sewerage networks,” explains Matto, who cites obstacles to improving the networks in urban areas that include the massive cost and disruption such work involves.

This leaves cities primarily dependent on on-site sanitation systems where toilets are usually connected to septic tanks. Installed and operated effectively, on-site systems offer a viable way of dealing with sewage, but, according to Matto, they often are not correctly installed and operated. “In many of the cities in India, the septic tanks do not meet Indian Standard 2470 code for installation, they are just sewage containers,” she says.

Even when the septic tanks and pit latrines are processing the waste ‘scientifically’, they generate sludge. Safely treated, this can be used as a fertilizer, but according to Matto it is often just dumped or stored. “SBM 2.0, launched in 2021, is making the processing of sludge a priority, including extraction of resources as part of the circular economy,” she points out.

WaterAid, an international not-for-profit organization focused on water, sanitation and hygiene (WASH) has made faecal sludge treatment a priority in India, supporting the installation of faecal sludge treatment plants serving both urban and rural households that are being managed by local authorities.

“The lessons learned on the projects are being shared through the Rural WASH Partners Forum and have laid the foundation for similar projects,” says Biswanath Sinha, director of policy and technical support in India for WaterAid, adding that some 2600 faecal sludge treatment plants are currently being built across the sub-continent.

There has also been work on new kinds of treatment systems that do all the processing on site. These include ‘omni processors’ that treat faecal sludge to remove pathogens while simultaneously extracting by-products with commercial value. Such projects have yet to take off in India because of their cost and maintenance requirements.

Medlicott welcomes innovative thinking and private sector involvement, but stresses the importance of government leadership. “Sanitation is a public good, and sanitation services need to be provided to everyone, with governments taking the lead where the market doesn’t reach,” she says.

In India, on-site treatment systems are typically installed and maintained by private companies, and this has not always served communities well. “Companies come in, build for a fee then move on,” says Matto.

One way to make sure this does not happen is for local authorities to pay the companies who install treatment facilities to maintain them. This approach is being used at the Lodhi Garden wastewater treatment project in New Delhi, where payment is dependent on the annual performance of the plant.

Medlicott believes tighter regulation can play a key role – citing data from over 120 countries which indicate that those with strong regulation are making faster progress towards the sustainable development goal (SDG) target for sanitation, which includes a specific target (6.2) for achieving access to adequate sanitation and ending open defecation by 2030.

India has regulation covering sewage treatment dating back to the Water Prevention and Control of Pollution Act of 1974, but enforcement has varied, reflecting local authority engagement and available resources.

Matto believes there is scope for greater community participation, noting several examples, among which initiatives supported by the state of Odisha where, as part of an inclusivity agenda, many of the treatment facilities are being operated and managed by transgender communities organized into self-help groups.

WaterAid is also supporting community-based programmes with local partners, including the formation of microcredit-linked self-help groups, promoting toilet construction across villages in Uttar Pradesh.

Despite the size of the obstacles to progress, Matto sees reasons for optimism, notably regarding open defecation. “Lesson have been learnt,” she says. But she has no illusions about the challenges faced, notably those presented by climate change. 

At the time of writing, her home city, Delhi – having just undergone an intense heatwave and severe water shortages – was being inundated by extreme rainfall which was impacting the city’s sanitation systems. “Drought is a problem but so is flooding,” she says.

Sekhar Raghaven could not agree more. Director of the Rain Centre, a Chennai-based civil society organization focused on rainwater harvesting and ecological sanitation systems, he lived through the much-publicized drought of 2019 which was followed by severe flooding in 2021. Both impacted the city’s water supply and sanitation system, but in ways that Raghaven believes have not been fully appreciated. 

“It was the wealthy who suffered most because their water was piped in from the city’s depleted reservoirs, whereas the urban poor continued to tap the city’s saline aquifers. Everyone was thirsty, but the poor could at least flush their toilets. A certain amount of resilience was built in.” 

As to whether lessons are being learnt, Raghaven is not entirely convinced. “More needs to be done, both in terms of rainwater harvesting and wastewater disposal,” he says.

Delegates at the Global Sanitation Summit organized by UNICEF, WHO and WaterAid which took place in Kathmandu, Nepal in June would probably agree.  The summit concluded that achieving SDG target 6.2 by 2030 is going to require a five-fold increase in efforts.

**Figure:**
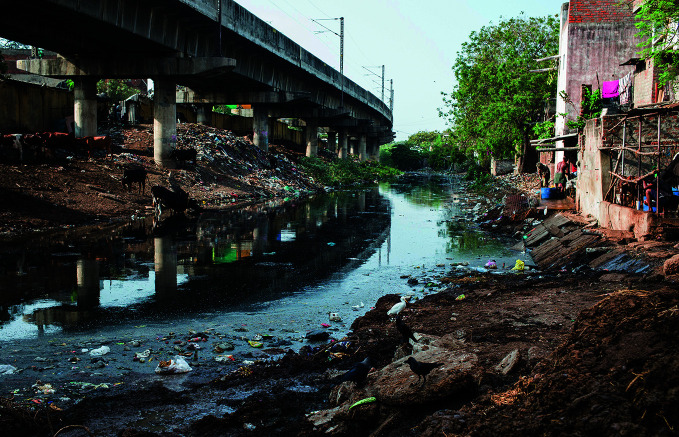
The banks of the Buckingham Canal, Chennai, India

WHO/Florian Lang

**Figure:**
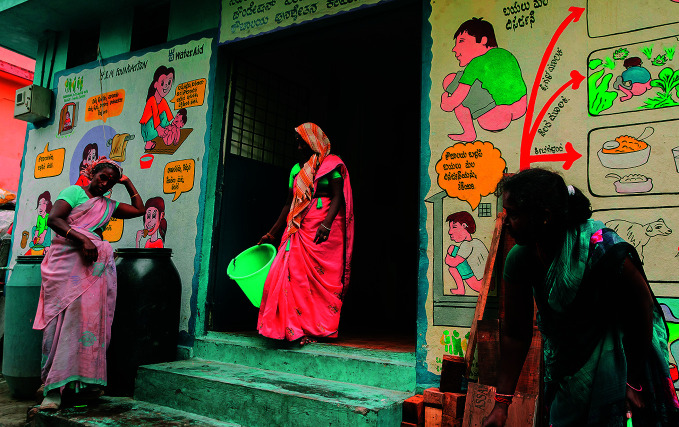
Retrofitted public toilet in Bengaluru, Karnataka, India

WaterAid

